# Comprehensive validation of fasting-based and oral glucose tolerance test–based indices of insulin secretion against gold standard measures

**DOI:** 10.1136/bmjdrc-2022-002909

**Published:** 2022-09-13

**Authors:** Katsiaryna Prystupa, Rebecka Renklint, Youssef Chninou, Julia Otten, Louise Fritsche, Sebastian Hoerber, Andreas Peter, Andreas L Birkenfeld, Andreas Fritsche, Martin Heni, Robert Wagner

**Affiliations:** 1 Department of Internal Medicine IV, Division of Diabetology, Endocrinology and Nephrology, Eberhard Karls Universität Tübingen, Tubingen, Germany; 2 Institute for Clinical Diabetology, German Diabetes Center, Leibniz Center for Diabetes Research at Heinrich Heine University, Düsseldorf, Germany; 3 German Center for Diabetes Research (DZD), Neuherberg, Germany; 4 Institute for Diabetes Research and Metabolic Diseases of the Helmholtz Center Munich at the University of Tübingen, Tubingen, Germany; 5 Department of Public Health and Clinical Medicine, Umea University, Umea, Sweden; 6 Institute for Clinical Chemistry and Pathobiochemistry, Department for Diagnostic Laboratory Medicine, University Hospital of Tübingen, Tubingen, Germany; 7 Division of Endocrinology and Diabetology, Department of Internal Medicine I, Ulm University Hospital, Ulm, Germany; 8 Medical Faculty, Department of Endocrinology and Diabetology, Heinrich Heine University, Düsseldorf, Germany

**Keywords:** insulin secretion, insulin, C-peptide

## Abstract

**Introduction:**

With pre-diabetes and diabetes increasingly recognized as heterogeneous conditions, assessment of beta-cell function is gaining clinical importance to identify disease subphenotypes. Our study aims to comprehensively validate all types of surrogate indices based on oral glucose tolerance test (OGTT) and fasting measurements in comparison with gold standard methods.

**Research design and methods:**

The hyperglycemic clamp extended with glucagon-like peptide 1 (GLP-1) infusion and intravenous glucose tolerance test (IVGTT), as well as OGTT, was performed in two well-phenotyped cohorts. The gold standard–derived indices were compared with surrogate insulin secretion markers, derived from fasting state and OGTT, using both Pearson’s and Spearman’s correlation coefficients. The insulin-based and C-peptide-based indices were analyzed separately in different groups of glucose tolerance and the entire cohorts.

**Results:**

The highest correlation coefficients were found for area under curve (AUC) (I_0-30_)/AUC (G_0-30_), I_30_/G_30_, first-phase Stumvoll and Kadowaki model. These indices have high correlation coefficients with measures obtained from both insulin and C-peptide levels from IVGTT and hyperglycemic clamp. AUC (I_0-120_)/AUC (G_0-120_), BIGTT-AIR_0-60-120_, I_30_/G_30_, first-phase Stumvoll and AUC (I_0-30_)/AUC (G_0-30_) demonstrated the strongest association with incretin-stimulated insulin response.

**Conclusions:**

We have identified glucose-stimulated and GLP-1-stimulated insulin secretion indices, derived from OGTT and fasting state, that have the strongest correlation with gold standard measures and could be potentially used in future researches and clinical practice.

**WHAT IS ALREADY KNOWN ON THIS TOPIC**

- Hyperglycemic clamp and intravenous glucose tolerance test (IVGTT) are considered gold standards to assess insulin secretion, but they are expensive and laborious.
- Several fasting-derived and oral glucose tolerance test (OGTT)-derived indices have been proposed and separately evaluated, but large head-to-head studies validating them to gold standard measurements are still lacking.

**WHAT THIS STUDY ADDS**

- Area under curve (AUC) (I_0-30_)/AUC (G_0-30_), I_30_/G_30_, first-phase Stumvoll and Kadowaki model showed the highest correlation coefficients with the gold standard measures.
- AUC (I_0-120_)/AUC (G_0-120_), BIGTT-AIR_0-60-120_, I_30_/G_30_, first-phase Stumvoll and AUC (I_0-30_)/AUC (G_0-30_) are the most reliable measures for evaluating glucagon-like peptide 1-stimulated insulin release.

**HOW THIS STUDY MIGHT AFFECT RESEARCH, PRACTICE OR POLICY**

- The use of robust OGTT-based indices enables an accurate assessment of insulin secretion, also contributing to pre-diabetes subphenotyping, the prediction of type 2 diabetes and its complications.

## Introduction

Reduced sensitivity to the action of insulin and deficient insulin secretion are two physiological defects underlying type 2 diabetes (T2D) and impaired glucose tolerance (IGT).

Insulin secretion and sensitivity are associated across a negative feedback loop, in which beta-cells reimburse for changes in whole-body insulin sensitivity through a proportional and reciprocal increase in insulin secretion.^1^

Insulin sensitivity and insulin secretion can be accurately determined by hyperinsulinemic-euglycemic and hyperglycemic clamps or intravenous glucose tolerance tests (IVGTTs) that are considered “gold standards” for these measurements.^2 3^ Precise assessment of these two traits is crucial to identify subphenotypes of pre-diabetes and T2D, which is gaining increasing importance by acknowledgment of the pathophysiological heterogeneity of this condition.^4 5^ Furthermore, these measures can support the prediction of diabetes in non-diabetic subjects.^6 7^ However, the invasive, expensive, and time-consuming gold standard procedures are not applicable in routine clinical practice.

Several indices have been proposed to estimate insulin sensitivity and insulin secretion, based on more readily measurable parameters obtained in the fasting state or after an oral glucose tolerance test (OGTT).^8^ Their usefulness is influenced by the degree of correlation with gold standard indicators, by their reproducibility^9^ and by their ability to predict diabetes incidence similarly to more complex methods.^10–12^

Many surrogate methods for insulin sensitivity assessment have been proposed and validated,^10 12^ but comprehensive studies to compare insulin secretion measures to gold standard methods are scarce. While several indices for beta-cell assessment based on dynamic changes in insulin and glucose during OGTT have been separately evaluated in numerous studies, most of these indices have not been compared head-to-head and there is still disagreement about their validity.^13–15^ Therefore, our work is aimed at performing a large, comprehensive comparative analysis of all types of indicators obtained in the fasting state or during OGTT to determine the secretion of insulin in comparison with the gold standards IVGTT and hyperglycemia clamp in two well-phenotyped cohorts. Moreover, the novel hyperglycemic clamp was used in one of our included trials^16^ allows for defining surrogate indices for glucagon-like peptide 1 (GLP1)–stimulated insulin secretion.

## Research design and methods

### Participants

The 392 subjects included in the present analysis were part of the Tuebingen Family Study (TUEF), who were recruited based on their increased risk for T2D (prior gestational diabetes, overweight or positive family history). Three hundred and sixteen subjects participated in OGTT and IVGTT, and 76 in OGTT and hyperglycemic clamps. Informed written consent to the studies was obtained from all participants. The studies adhered to the Declaration of Helsinki. The study protocols were approved by the Ethical Committee of the Medical Faculty of the University of Tübingen (422/2002).

All participants were classified into different stages of glucose tolerance according to the revised WHO criteria.^17^ The IVGTT cohort consists of 209 subjects with normal glucose tolerance (NGT), 91 with impaired fasting glucose (IFG) and/or IGT, and 16 with test-diagnosed T2D (table 1).

**Table 1:** Clinical and biochemical characteristics of the study population

Participated in OGTT and IVGTT	
n	316
Sex, male/female	183/133
Age (median (IQR))	47.00 (37.00–54.00)
BMI (median (IQR))	28.59 (25.11–32.20)
NGT (%)	209 (66.1%)
IFG and/or IGT	91 (28.8%)
Type 2 diabetes	16 (5.1%)
**Participated in OGTT and hyperglycemic clamp**	
n	76
Sex, male/female	33/43
Age (median (IQR))	36.00 (26.00–46.25)
BMI (median (IQR))	24.48 (22.01–26.94)
NGT	49 (64.5%)
IFG and/or IGT	23 (30.2%)
Type 2 diabetes	4 (5.3%)

BMI, body mass index; IFG, impaired fasting glucose; IGT, impaired glucose tolerance; IVGTT, intravenous glucose tolerance test; NGT, normal glucose tolerance; OGTT, oral glucose tolerance test.

Of the 91 subjects with IFG/IGT, 28 had isolated IFG (fasting plasma glucose 6.1–6.9 mmol/L), 40 had isolated IGT (2-hour glucose value 7.8–11.0 mmol/L), 23 had both IFG and IGT, and 16 had newly diagnosed T2D. The hyperglycemic clamp group comprises 49 NGT, 23 pre-diabetes (IFG and/or IGT), and 4 screen-diagnosed T2D participants.

### Procedures and calculations

In the IVGTT, an intravenous bolus injection of glucose was given (0.3 g/kg body weight in a 20% solution) at time 0 after baseline blood draw. Blood was sampled at 0, 2, 4, 6, 8, 10, 20, 30, 40, 50, and 60 min for measuring plasma glucose, insulin, and C-peptide (CP) concentration.^18^ The incremental area under curve (iAUC_0-10_) for insulin and CP over the first 10 min of the test was determined using the trapezoidal method. Multivariate imputation by chained equations^19^ was performed for missingness, which was less than 2.6% and completely random.

The hyperglycemic clamp was performed as previously described.^16^ In brief, the clamp was initiated with a weight-adapted intravenous bolus of 20% glucose over 1 min and continued with an infusion of 20% glucose with periodic adjustments based on the negative feedback principle to maintain blood glucose at 10 mmol/L. After 120 min, GLP-1 was given as a bolus injection (4.5 pmol/kg), followed by continuous infusion of 1.5 pmol/kg/min during the next 60 min. Serum samples for insulin, CP, and glucose were drawn at −30 to –15, 0, 2.5, 5, 7.5, 10, 15, 30, 60, 90, 120, 140, 160, 170, and 180 min. Insulin secretion after an arginine bolus given at the end of the clamp was not analyzed in the current work. First phase of insulin release, reflecting the early insulin peak secreted from the pancreatic beta-cell in response to glucose stimulation, was calculated as the sum of insulin or CP measurements at 2.5, 5, 7.5, and 10 min. The second phase of glucose-stimulated insulin release was derived as means of insulin or CP at 80, 100, and 120 min. GLP-1-stimulated insulin (CP) secretion at 10 mM glucose was assessed as a mean of 160, 170, and 180 min.

CP and insulin concentrations were determined using chemiluminescent methods on an ADVIA Centaur XPT analyzer (Siemens Healthineers, Eschborn, Germany). Glucose concentrations were measured using a hexokinase method on an ADVIA clinical chemistry XPT analyzer (Siemens Healthineers). The limits of quantification of the insulin and CP assays are 1 pmol/L and 16 pmol/L, respectively. Using quality control samples for the determination of assay imprecision, typical coefficients of variation were obtained: 4.6% (target concentration: 136 pmol/L) and 5.2% (597 pmol/L) using the insulin assay and 5.8% (337 pmol/L) and 6.2% (1470 pmol/L) using the CP assay.

All 392 persons participated in the OGTT, taking 75 g of glucose in a volume of 300 mL after an overnight fast. Samples for glucose and insulin measurements were taken at 0, 30, 60, and 120 min. Insulin secretion indices were calculated from these OGTTs.

The insulinogenic index (IGI), an index of beta-cell function, was computed as (I_30_−I_0_)/(G_30_−G_0_), (I_60_−I_0_)/(G_60_−G_0_), and (I_120_−I_0_)/(G_120_−G_0_), with I_n_, G_n_, plasma concentrations at the nth minute for insulin and glucose, respectively.^15^

The beta-cell function insulin sensitivity glucose tolerance test (BIGTT) method is an estimation of an acute insulin response (AIR) derived from the following equation: BIGTT-AIR_0-30-120_ = exp[8.20+(0.00178×I_0_)+(0.00168×I_30_)-(0.000383×I_120_)−(0.314×G_0_)−(0.109×G_30_)+(0.0781×G_120_)+(0.180×sex)+(0.032×BMI)], BIGTT-AIR_0-60-120_=exp[8.20+(0.00178×I_0_)+(0.00168×I_60_)−(0.000383×I_120_)−(0.314×G_0_)−(0.109×G_60_)+(0.0781×G_120_)+(0.180×sex)+(0.032×BMI)].^20^

The ratio of the AUC for insulin and for CP to AUC for glucose over a specified time frame was calculated by applying the trapezoid rule (AUC (I_all_), AUC (CP_all_), AUC (I_0-30_)/AUC (G_0-30_), AUC (CP_0-30_)/AUC (G_0-30_), AUC (CP_0-60_)/AUC (G_0-60_), AUC(I_0-60_)/AUC(G_0-60_), AUC(I_0-120_)/AUC (G_0-120_), AUC (CP_0-120_)/AUC (G_0-120_)).^14^ The additional indices of insulin secretion used in this study were as follows: the corrected insulin response (CIR)=I_30_/(G_30_×(G_30_−3.89)), I_60_/(G_60_×(G_60_−3.89)), I_120_/(G_120_×(G_120_−3.89))^13^; the insulin/glucose ratio derived as I_0_/G_0_, I_30_/G_30_, I_60_/G_60_, and I_120_/G_120_, CP/glucose ratio CP_0_/G_0_ and CP_120_/G_120_, insulin and CP ratio at minute 30 (I_30_/I_0_, CP_30_/CP_0_), minute 60 (I_60_/I_0_, CP_60_/CP_0_) and at minute 120 (I_120_/I_0_, CP_120_/CP_0_), delta insulin at 30 min ΔI_30_=I_30_−I_0_ and 60 min ΔI_60_=I_60_−I_0_, CP and insulin at fasting state (CP_0_, I_0_), minute 30 (CP_30_, I_30_), minute 60 (CP_60_, I_60_), and minute 120 (CP_120_, I_120_)^13 21^; first-phase Stumvoll=1283+1.829×I_30_−138.7×G_30_+3.772×I_0_; and second-phase Stumvoll=286+0.416×I_30_−25.94×G_30_+0.926×I_0_^11^; Kadowaki model=(I_30_−I_0_)/(G_30_−G_0_)^22^; C-peptide index (CPI) CPI_120_=100×C_P120_(ng/mL)/G_120_(mg/dL), CPI_0_=100×C_P0_(ng/mL)/G_0_(mg/dL)^23^; log-transformed insulin at minute 0, 30, 60, 90, 120 (log(I_0_), log(I_30_), log(I_60_), log(I_90_), log(I_120_)).^24^

The Homeostasis Model Assessment (HOMA)−%B was calculated using the fasting plasma insulin and glucose concentration (HOMA−%B=(20×I_0_)/(G_0_−3.5)).^25^

Fasting CP, insulin, and glucose levels were used in the homeostasis model assessment computer model to generate estimates of beta-cell function (HOMA2%B).^25^

### Statistical analysis

Statistical analyses were performed with R (software V.4.0.3).^26^ Descriptive data are expressed as medians±IQRs. The relationship between the different estimates of insulin secretion was determined using both Pearson’s and Spearman’s correlation coefficients. The insulin and CP-derived insulin secretion indices were separately analyzed. The best-performing indices were selected according to Pearson’s correlation coefficients, and a high correlation was defined as 0.6 or more. P values <0.05 were considered statistically significant.

## Results

Our analysis included all participants of the TUEF study from whom hyperglycemic clamp or IVGTT and OGTT were available (n=392). We analyzed data from 316 persons (133 men and 183 women), who underwent IVGTTs and OGTTs, as well as 76 (33 men and 43 women) in whom hyperglycemic clamp and OGTT were performed. The characteristics of the study population are summarized in table 1.

### Comparison of insulin secretion indices with dynamic insulin secretion measurements by IVGTT

The insulin secretion indices obtained from fasting or OGTT measurements were tested against IVGTT-obtained measurements (insulin iAUC_0-10_ and CP iAUC_0-10_) of beta-cell capacity.

The vast majority of tested surrogate insulin secretion indices except for CP_120_/CP_0_ and I_120_/I_0_ correlated significantly with first-phase insulin and CP-based IVGTT values in the group of NGT as well as in IFG and/or IGT group (online supplemental table 1). In NGT, the strongest correlation with first-phase insulin and CP by both Spearman’s and Pearson’s methods was found for CIR_30_, I_30_/G_30_, BIGTT-AIR_0-30-120_, first-phase Stumvoll, and AUC (I_0-30_)/AUC (G_0-30_) (online supplemental table 1).

10.1136/bmjdrc-2022-002909.supp1 Supplementary data

In pre-diabetes, the strongest correlation between insulin iAUC_0-10_ and CP iAUC_0-10_ was identified for AUC (CP_0-60_)/AUC (G_0-60_), CP_30_, CP_60_/G_60_, and CP_30_/G_30_ (online supplemental table 1).

The highest correlation in the entire cohort was detected for AUC (I_0-30_)/AUC (G_0-30_), first-phase Stumvoll, Kadowaki model, IGI_30_, CIR_30_, and I_30_/G_30_. The weakest correlation for both groups had CIR_120_ and IGI_120_.

We also analyzed the correlation coefficients in a group of participants with test-diagnosed T2D (n=16). According to our analysis, CP_30_/CP_0_, CP_60_/CP_0_, CIR_30_, I_30_/I_0_, and BIGTT-AIR_0-30-120_ correlated significantly with both insulin iAUC_0-10_ and CP iAUC_0-10_ (online supplemental table 1).

### Comparison of insulin secretion indices with dynamic insulin secretion measurements in the hyperglycemic clamp

We tested different formulas for calculating insulin secretion from OGTT and hyperglycemic clamp techniques.

An overwhelming majority of surrogate indices have a positive correlation with the first-phase insulin response obtained from the gold standard in participants with NGT as well as with IFG or glucose tolerance (online supplemental table 2). In NGT, the numerically highest significant correlation with clamp-derived first-phase insulin secretion measured by insulin and CP was detected for AUC (I_0-30_)/AUC (G_0-30_), I_30_, I_30_/G_30_, and BIGTT-AIR_0-30-120_ (online supplemental table 2).

According to both correlation coefficients, the strongest relationship between first-phase clamp-derived and OGTT-derived measurements in IFG/IGT group correspond to AUC (I_0-30_)/AUC (G_0-30_), I_30_/G_30_, BIGTT-AIR_0-30-120_, and Kadowaki model (online supplemental table 2).

The second-phase insulin and CP strongly correlate with AUC (I_0-30_)/AUC (G_0-30_), AUC (I_0-120_)/AUC (G_0-120_), I_30_, I_30_/G_30_, BIGTT-AIR_0-30-120_ in NGT (online supplemental table 2) and with I_0_/G_0_, AUC (I_0-30_)/AUC (G_0-30_), BIGTT-AIR_0-30-120_, I_30_/G_30_, first-phase Stumvoll in pre-diabetes (online supplemental table 2).

The highest correlation coefficients with first-phase and second-phase insulin secretion are shown by AUC (I_0-30_)/AUC (G_0-30_), BIGTT-AIR_0-30-120_, I_30_/G_30_, I_30_, and first-phase Stumvoll. The surrogate measures with the lowest correlation are CP_120_, log(I_120_) and log(I_90_).

Due to the small number of participants with test-diagnosed T2D (n=4), they were excluded from this analysis.

**Figure 1:**
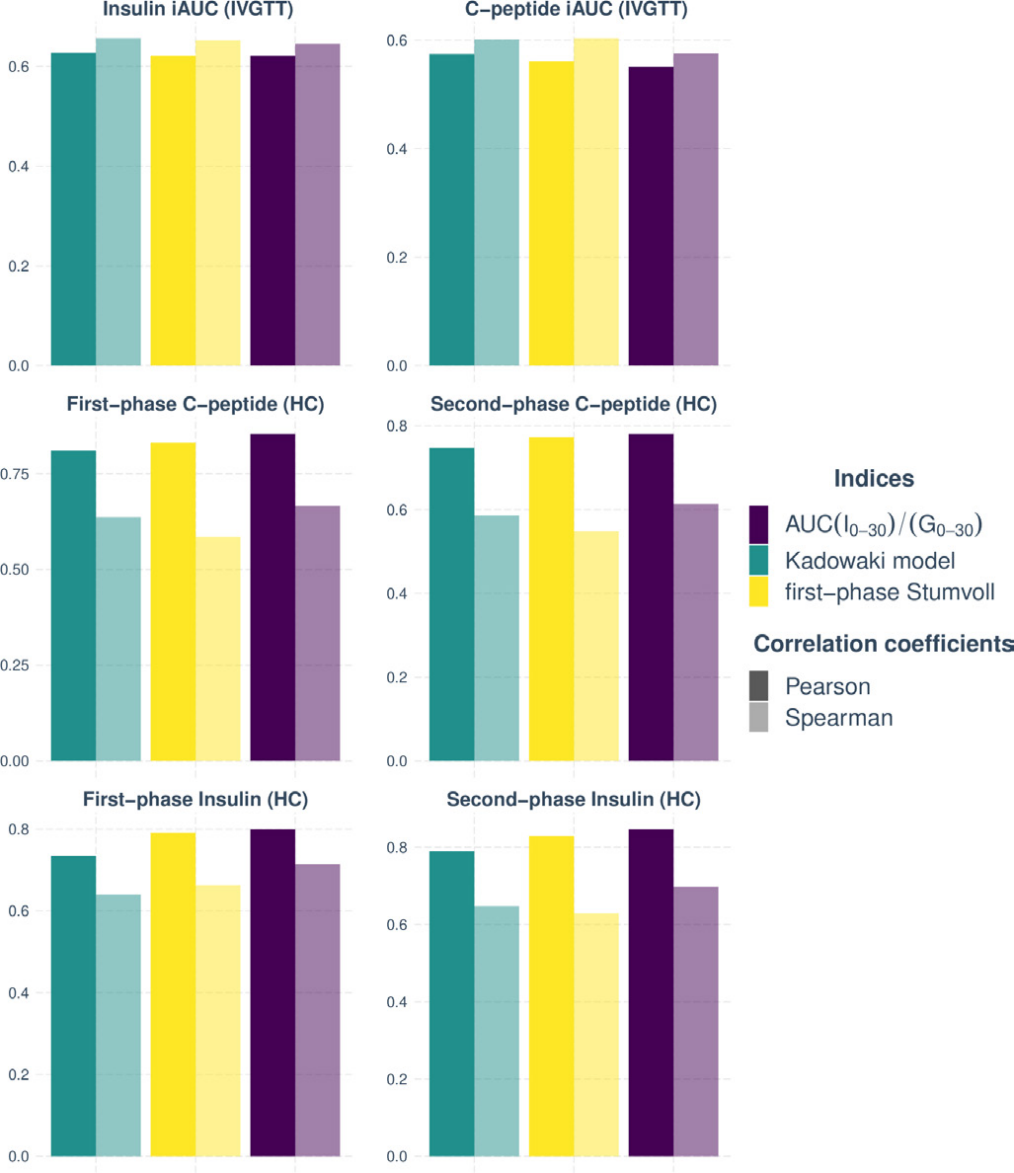
Indices with the highest Spearman’s and Pearson’s correlation coefficients in pre-diabetes and NGT groups in both study cohorts. HC, hyperglycemic clamp; iAUC, incremental area under curve; IVGTT, intravenous glucose tolerance test; NGT, normal glucose tolerance.

Comparison of surrogate indices measuring insulin secretion using OGTT with the gold standard criteria revealed three indices (I_30_/G_30_, AUC (I_0-30_)/AUC (G_0-30_), first-phase Stumvoll, and Kadowaki model) with the highest Pearson’s and Spearman’s coefficients for both groups (NGT and pre-diabetes) in two phases and during all tests (figure 1). However, the CIR_120_ and IGI_120_ are the overall indices with the lowest significant correlation compared with the insulin and CP gold standard measurements according to both correlations’ methods in the groups.

### GLP-1-induced insulin (C-peptide) secretion

**Figure 2:**
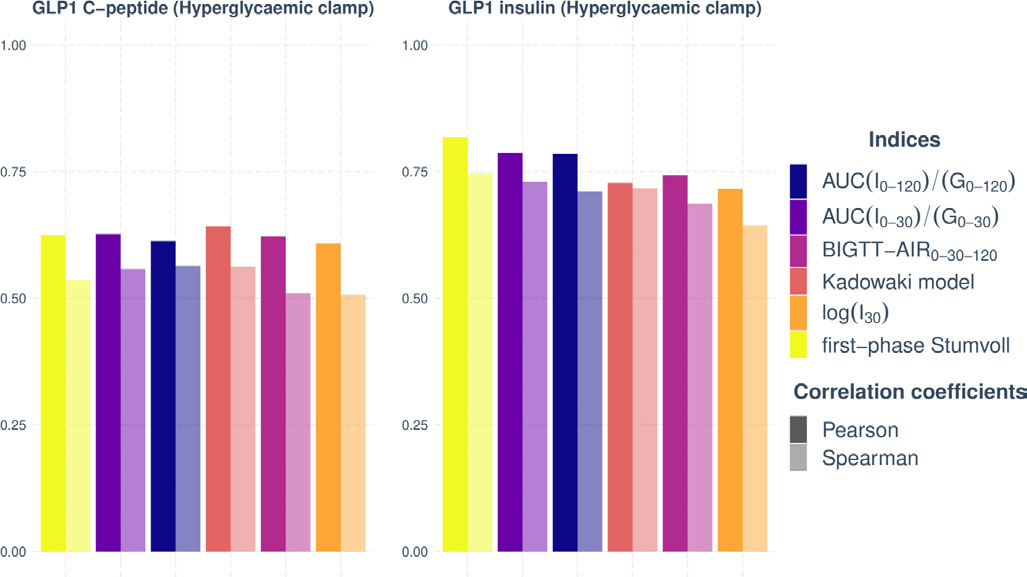
Top indices by Spearman’s and Pearson’s correlation coefficients in a group of 76 participants compared with GLP-1-stimulated insulin secretion during hyperglycemic clamp. AIR, acute insulin response; AUC, area under curve; BIGTT, beta-cell function insulin sensitivity glucose tolerance test; GLP-1, glucagon-like peptide 1.

Using data from the modified hyperglycemic clamp, with a GLP-1 infusion phase,^16^ we also tested the relationship between GLP-1-stimulated insulin release and OGTT-derived measurements. The vast majority of indices in NGT and pre-diabetes correlate significantly with clamp-derived GLP1-stimulated insulin secretion index. The strongest correlation belongs to I_30_/G_30_, first-phase Stumvoll, AUC (I_0-120_)/AUC (G_0-120_), AUC (I_0-30_)/AUC (G_0-30_), and BIGTT-AIR_0-60-120_ (figure 2). The weakest correlation coefficients have CIR_120_ and CPI_120_.

## Discussion

In this study, we compared fasting-based and/or OGTT-based insulin secretion markers to gold-standard measurements. Our study revealed that most surrogate indices of insulin secretion correlated significantly with dynamic measurements assessed from IVGTT and hyperglycemic clamp. The substantial difference between Pearson’s and Spearman’s correlation coefficients for some surrogate indices can be attributed to variables with skewed distributions.

The overall highest correlation coefficients calculated by both Pearson’s and Spearman’s tests were found for AUC (I_0-30_)/AUC (G_0-30_), I_30_/G_30_, first-phase Stumvoll, and Kadowaki model. These indices have high correlation coefficients with gold standard measures, obtained both from insulin and CP measurements, both with IVGTT and hyperglycemic clamp. All four indices reflect insulin release and comprise OGTT-derived insulin and glucose levels at minutes 0 and 30 and predict insulin secretion abnormalities better than 120-min-based indices (eg, CP_120_, CIR_120_, and IGI_120_). The somewhat weaker correlation with 120- min indices can probably be due to the changes in the incretin axis caused by oral intake of glucose.^27^ Our findings are consistent with previously published results from numerous studies that include these indices.^11 14 20 22 28^

The high correlation of AUC (I_0-120_)/AUC (G_0-120_) and BIGTT-AIR_0-60-120_ with GLP-1-stimulated insulin secretion in our analysis argues for a longer assessment during OGTT when addressing incretin effects. The strong relationship between incretin-stimulated response and AUC (I_0-30_)/AUC (G_0-30_), I_30_/G_30_, first-phase Stumvoll in all experiments stresses the robustness of these surrogate indices to estimate insulin secretion. Another possible contributor to a comparably better correlation of short-term insulin/CP assessment during OGTT with gold standard measures could be a diverging influence of insulin clearance over time.^9^

Parenteral and oral glucose loading leads to the stimulation of glucose with the involvement of various physiological processes. Oral glucose ingestion activates complex mechanisms, including an incretin-related cascade and even the brain^29^ that all in concert control insulin release.

The correlation between gold standard measures and insulin-based indices was higher than with CP-based indices. This is consistent with previous results.^15^ CP is considered to better reflect insulin release,^30 31^ but the vast majority of previously studied surrogate indices are based on measuring insulin. Due to a long half-life of CP (20–30 min), each measurement integrates insulin release from a more extensive time period, which not necessarily captures the aimed stimulated time frame.^15 25 32^

One limitation of this work arises from the lack of ethnic heterogeneity in studied population. The investigated measures may not necessarily work similarly well in other ethnicities such as persons of Asian origin, who often have lower beta-cell function,^33^ or persons of African origin, showing beta-cell hyper-responsiveness and decreased insulin clearance.^34^

Taken together, we here determined which indices can most reliably estimate insulin secretion from fasting measures or OGTT. Given the importance of assessing insulin secretion for the classification of patients in novel subgroups of pre-diabetes^5^ and diabetes,^4^ also for potential therapeutic considerations,^35^ selection of appropriate estimates from OGTTs is crucial in research and could also play a role in future clinical practice.

## Data Availability

Data are available on reasonable request. The data are not publicly available due to them containing information that could compromise research participant privacy/consent and may be made available upon reasonable request.
