# Supplementary material for: Comprehensive validation of fasting-based and oral glucose tolerance test–based indices of insulin secretion against gold standard measures
Source: BMJ Open Diabetes Res Care. 2022 Sep 13;10(5):e002909. doi: 10.1136/bmjdrc-2022-002909 (PMC9472162; doi:10.1136/bmjdrc-2022-002909)
Supplement: Supplementary data [file bmjdrc-2022-002909supp001.pdf]

Supplementary Material

Supplementary Table 1: Correlation between fasting and OGTT-derived insulin secretion indices and first-phase insulin secretion measured with the IVGTT. (* p<0.05; **p<0.01; ***p<0.001)

Fasting/OGTT-based indices	IVGTT-derived measurements											
	NGT Insulin $iAUC_{0-10}$ Spearman	NGT Insulin $iAUC_{0-10}$ Pearson	NGT C-peptide $iAUC_{0-10}$ Spearman	NGT C-peptide $iAUC_{0-10}$ Pearson	IFG/IGT Insulin $iAUC_{0-10}$ Spearman	IFG/IGT Insulin $iAUC_{0-10}$ Pearson	IFG/IGT C-peptide $iAUC_{0-10}$ Spearman	IFG/IGT C-peptide $iAUC_{0-10}$ Pearson	T2D Insulin $iAUC_{0-10}$ Spearman	T2D Insulin $iAUC_{0-10}$ Pearson	T2D C-peptide $iAUC_{0-10}$ Spearman	T2D C-peptide $iAUC_{0-10}$ Pearson
AUC (CP_{0-120})/AUC (G_{0-120})	0.428***	0.392***	0.475***	0.445***	0.573***	0.501***	0.551***	0.513***	0.296	0.22	0.136	0.222
AUC(CP_{0-30})/AUC(G_{0-30})	0.546***	0.556***	0.572***	0.58***	0.597***	0.555***	0.56***	0.544***	0.165	0.049	0.033	0.063
AUC (CP_{0-60})/AUC (G_{0-60})	0.403***	0.364***	0.443***	0.407***	0.591***	0.563***	0.564***	0.56***	0.236	0.172	0.046	0.144
AUC(CP_{all})	0.311***	0.297***	0.296***	0.3***	0.453***	0.426***	0.42***	0.407***	0.186	0.071	0.021	0.035
AUC(I_{all})	0.435***	0.439***	0.355***	0.373***	0.48***	0.355***	0.367***	0.252*	0.243	0.209	0.125	0.227
AUC(I_{0-120})/AUC(G_{0-120})	0.525***	0.534***	0.462***	0.48***	0.555***	0.405***	0.45***	0.31**	0.443	0.339	0.321	0.373
AUC(I_{0-30})/AUC(G_{0-30})	0.635***	0.665***	0.576***	0.604***	0.634***	0.513***	0.537***	0.424***	0.468	0.409	0.368	0.346
AUC(I_{0-60})/AUC(G_{0-60})	0.423***	0.399***	0.367***	0.379***	0.542***	0.465***	0.426***	0.378***	0.35	0.213	0.204	0.243
$BIGTT\text{-}AIR_{0-30-120}$	0.651***	0.697***	0.596***	0.619***	0.483***	0.411***	0.365***	0.295**	0.382	0.411	0.593*	0.525*
$BIGTT\text{-}AIR_{0-60-120}$	0.531***	0.54***	0.5***	0.534***	0.468***	0.51***	0.347***	0.424***	0.175	0.173	0.396	0.306
CIR_{120}	0.259***	0.129	0.255***	0.155*	0.459***	0.17	0.433***	0.198	0.582*	0.293	0.521*	0.381
CIR_{30}	0.646***	0.616***	0.642***	0.616***	0.565***	0.452***	0.496***	0.416***	0.668**	0.607*	0.579*	0.573*
CIR_{60}	0.43***	0.288***	0.447***	0.341***	0.598***	0.396***	0.518***	0.382***	0.493	0.33	0.354	0.346
CP_{120}/CP_0	-0.109	-0.13	-0.105	-0.118	-0.128	-0.169	-0.079	-0.11	0.196	0.436	0.218	0.411
CP_{30}/CP_0	0.264***	0.201**	0.301***	0.241***	0.321**	0.274**	0.366***	0.303**	0.684**	0.807***	0.556*	0.642*
CP_{60}/CP_0	-0.048	-0.092	-0.02	-0.07	0.112	0.102	0.183	0.156	0.529*	0.716**	0.264	0.49
CP_0	0.31***	0.342***	0.278***	0.323***	0.331**	0.345***	0.272**	0.294**	-0.129	-0.251	-0.182	-0.214

Fasting/OGTT-based indices	IVGTT-derived measurements											
	NGT Insulin $iAUC_{0-10}$ Spearman	NGT Insulin $iAUC_{0-10}$ Pearson	NGT C-peptide $iAUC_{0-10}$ Spearman	NGT C-peptide $iAUC_{0-10}$ Pearson	IFG/IGT Insulin $iAUC_{0-10}$ Spearman	IFG/IGT Insulin $iAUC_{0-10}$ Pearson	IFG/IGT C-peptide $iAUC_{0-10}$ Spearman	IFG/IGT C-peptide $iAUC_{0-10}$ Pearson	T2D Insulin $iAUC_{0-10}$ Spearman	T2D Insulin $iAUC_{0-10}$ Pearson	T2D C-peptide $iAUC_{0-10}$ Spearman	T2D C-peptide $iAUC_{0-10}$ Pearson
CP_0/G_0	0.314***	0.355***	0.294***	0.344***	0.356***	0.367***	0.293**	0.319**	-0.007	-0.12	-0.054	-0.018
CP_{120}	0.183**	0.156*	0.164*	0.152*	0.277**	0.213*	0.249*	0.206	0.029	0.044	-0.032	0.059
CP_{120}/G_{120}	0.203**	0.179**	0.225**	0.21**	0.452***	0.312**	0.423***	0.326**	0.214	0.194	0.204	0.26
CP_{30}	0.52***	0.484***	0.523***	0.496***	0.614***	0.578***	0.575***	0.56***	0.059	0.063	-0.147	-0.039
CP_{30}/G_{30}	0.57***	0.555***	0.609***	0.593***	0.629***	0.566***	0.613***	0.576***	0.314	0.189	0.138	0.146
CP_{60}	0.291***	0.26***	0.273***	0.264***	0.485***	0.513***	0.466***	0.503***	0.236	0.103	0.001	0.009
CP_{60}/G_{60}	0.402***	0.334***	0.47***	0.401***	0.604***	0.561***	0.593***	0.578***	0.357	0.258	0.132	0.185
HOMA-%B	0.439***	0.483***	0.357***	0.417***	0.452***	0.421***	0.309**	0.308**	-0.139	-0.193	0.029	0.088
HOMA2-%B(CP)	0.286***	0.313***	0.284***	0.32***	0.382***	0.369***	0.323**	0.333**	0.107	0.057	0.186	0.228
HOMA2-%B(I)	0.401***	0.475***	0.333***	0.404***	0.421***	0.419***	0.282**	0.308**	-0.077	-0.144	0.218	0.211
I_0	0.456***	0.499***	0.356***	0.402***	0.418***	0.427***	0.296**	0.326**	-0.368	-0.47	-0.286	-0.319
I_0/G_0	0.468***	0.517***	0.375***	0.425***	0.438***	0.446***	0.308**	0.343***	-0.307	-0.372	-0.154	-0.151
I_{120}	0.322***	0.297***	0.254***	0.228***	0.289**	0.232*	0.206	0.134	0.075	0.191	0.036	0.226
I_{120}/G_{120}	0.352***	0.339***	0.296***	0.28***	0.385***	0.272**	0.308**	0.19	0.325	0.287	0.282	0.345
I_{30}	0.586***	0.582***	0.519***	0.524***	0.63***	0.49***	0.535***	0.401***	0.404	0.358	0.239	0.208
ΔI_{30}	0.579***	0.567***	0.517***	0.515***	0.63***	0.478***	0.539***	0.397***	0.493	0.464	0.279	0.279
I_{30}/G_{30}	0.645***	0.682***	0.594***	0.627***	0.634***	0.513***	0.548***	0.435***	0.546*	0.511	0.357	0.399
I_{60}	0.326***	0.316***	0.255***	0.288***	0.468***	0.423***	0.372***	0.355***	0.268	0.148	0.107	0.132
ΔI_{60}	0.3***	0.288***	0.235***	0.267***	0.46***	0.413***	0.373***	0.349***	0.332	0.209	0.132	0.173
I_{60}/G_{60}	0.434***	0.401***	0.395***	0.393***	0.562***	0.473***	0.458***	0.394***	0.389	0.266	0.196	0.267
IGI_{120}	0.222**	0.136	0.164*	0.109	0.43***	0.205	0.363**	0.162	0.196	0.164	0.174	0.206
IGI_{30}	0.665***	0.583***	0.655***	0.594***	0.606***	0.495***	0.528***	0.445***	0.682**	0.608*	0.432	0.428
IGI_{60}	0.406***	0.184**	0.423***	0.246***	0.544***	0.271**	0.445***	0.266*	0.454	0.296	0.196	0.252
I_{120}/I_0	-0.071	-0.033	-0.065	-0.022	-0.068	-0.097	-0.057	-0.075	0.379	0.601*	0.336	0.521*

**Fasting/OGTT-based indices**	**IVGTT-derived measurements**											
	**NGT Insulin $iAUC_{0-10}$ Spearman**	**NGT Insulin $iAUC_{0-10}$ Pearson**	**NGT C-peptide $iAUC_{0-10}$ Spearman**	**NGT C-peptide $iAUC_{0-10}$ Pearson**	**IFG/IGT Insulin $iAUC_{0-10}$ Spearman**	**IFG/IGT Insulin $iAUC_{0-10}$ Pearson**	**IFG/IGT C-peptide $iAUC_{0-10}$ Spearman**	**IFG/IGT C-peptide $iAUC_{0-10}$ Pearson**	**T2D Insulin $iAUC_{0-10}$ Spearman**	**T2D Insulin $iAUC_{0-10}$ Pearson**	**T2D C-peptide $iAUC_{0-10}$ Spearman**	**T2D C-peptide $iAUC_{0-10}$ Pearson**
I_{30}/I_0	0.255***	0.193**	0.272***	0.242***	0.248*	0.164	0.284**	0.188	0.611*	0.642**	0.382	0.376
I_{60}/I_0	-0.078	-0.109	-0.053	-0.066	0.111	0.096	0.125	0.125	0.843***	0.662**	0.425	0.381
Kadowaki model	0.635***	0.662***	0.588***	0.613***	0.629***	0.501***	0.548***	0.433***	0.689**	0.609*	0.489	0.453
$\log(I_0)$	0.456***	0.491***	0.356***	0.398***	0.431***	0.436***	0.298**	0.333**	-0.368	-0.424	-0.286	-0.252
$\log(I_{120})$	0.322***	0.325***	0.254***	0.257***	0.287**	0.207*	0.206	0.153	0.075	0.133	0.036	0.187
$\log(I_{30})$	0.586***	0.572***	0.519***	0.523***	0.63***	0.56***	0.535***	0.493***	0.404	0.357	0.239	0.219
$\log(I_{60})$	0.326***	0.305***	0.255***	0.267***	0.479***	0.435***	0.371***	0.368***	0.268	0.13	0.107	0.13
$\log(I_{90})$	0.204**	0.209**	0.133	0.132	0.307**	0.238*	0.184	0.153	0.264	0.091	0.204	0.222
CPI_0	0.314***	0.355***	0.294***	0.344***	0.356***	0.367***	0.293**	0.319**	-0.007	-0.12	-0.054	-0.018
CPI_{120}	0.203**	0.179**	0.225**	0.21**	0.452***	0.312**	0.423***	0.326**	0.214	0.194	0.204	0.26
first-phase Stumvoll	0.658***	0.686***	0.615***	0.631***	0.591***	0.495***	0.498***	0.406***	0.461	0.43	0.511	0.473
second-phase Stumvoll	0.561***	0.574***	0.479***	0.504***	0.608***	0.494***	0.497***	0.392***	0.179	0.056	0.082	-0.028

Supplementary Table 2: Correlation between fasting and OGTT-derived insulin secretion indices and first/second-phase insulin secretion measured with the hyperglycaemic clamp. (* p<0.05; **p<0.01; ***p<0.001)

Fasting/OGTT-based indices	Hyperglycaemic clamp – derived measurements															
	NGT Insulin $1^{\text{st-phase}}$ Spearman	NGT Insulin $1^{\text{st-phase}}$ Pearson	NGT C-peptide $1^{\text{st-phase}}$ Spearman	NGT C-peptide $1^{\text{st-phase}}$ Pearson	NGT Insulin $2^{\text{d-phase}}$ Spearman	NGT Insulin $2^{\text{d-phase}}$ Pearson	NGT C-peptide $2^{\text{d-phase}}$ Spearman	NGT C-peptide $2^{\text{d-phase}}$ Pearson	IFG/IGT Insulin $1^{\text{st-phase}}$ Spearman	IFG/IGT Insulin $1^{\text{st-phase}}$ Pearson	IFG/IGT C-peptide $1^{\text{st-phase}}$ Spearman	IFG/IGT C-peptide $1^{\text{st-phase}}$ Pearson	IFG/IGT Insulin $2^{\text{d-phase}}$ Spearman	IFG/IGT Insulin $2^{\text{d-phase}}$ Pearson	IFG/IGT C-peptide $2^{\text{d-phase}}$ Spearman	IFG/IGT C-peptide $2^{\text{d-phase}}$ Pearson
AUC (CP_{0-120})/AUC (G_{0-120})	0.508***	0.694***	0.542***	0.566***	0.585***	0.505***	0.578***	0.59***	0.605**	0.589**	0.659***	0.655***	0.457*	0.61**	0.527*	0.618**
AUC(CP_{0-30})/AUC(G_{0-30})	0.49***	0.733***	0.524***	0.655***	0.518***	0.557***	0.5***	0.624***	0.768***	0.788***	0.757***	0.833***	0.64**	0.802***	0.617**	0.756***
AUC (CP_{0-60})/AUC (G_{0-60})	0.579***	0.711***	0.612***	0.611***	0.593***	0.564***	0.61***	0.619***	0.63**	0.696***	0.633**	0.729***	0.502*	0.695***	0.486*	0.646**
AUC(CP_{all})	0.45**	0.63***	0.519***	0.566***	0.648***	0.566***	0.664***	0.643***	0.657**	0.477*	0.646**	0.575**	0.527*	0.505*	0.45*	0.579**
AUC(I_{all})	0.593***	0.728***	0.52***	0.788***	0.746***	0.837***	0.605***	0.751***	0.582**	0.51*	0.517*	0.611**	0.549**	0.599**	0.397	0.693***
AUC(I_{0-120})/AUC(G_{0-120})	0.65***	0.857***	0.577***	0.852***	0.785***	0.887***	0.646***	0.809***	0.657***	0.619**	0.596**	0.704***	0.598**	0.699***	0.511*	0.755***
AUC(I_{0-30})/AUC(G_{0-30})	0.764***	0.913***	0.666***	0.896***	0.748***	0.902***	0.593***	0.766***	0.704***	0.825***	0.639**	0.852***	0.624**	0.855***	0.611**	0.838***
AUC(I_{0-60})/AUC(G_{0-60})	0.596***	0.665***	0.509***	0.733***	0.711***	0.779***	0.579***	0.678***	0.649**	0.67***	0.583**	0.735***	0.628**	0.725***	0.514*	0.757***

Fasting/OGTT-based indices	Hyperglycaemic clamp – derived measurements															
	NGT Insulin $1^{st\text{-}phase}$ Spearman	NGT Insulin $1^{st\text{-}phase}$ Pearson	NGT C-peptide $1^{st\text{-}phase}$ Spearman	NGT C-peptide $1^{st\text{-}phase}$ Pearson	NGT Insulin $2^{d\text{-}phase}$ Spearman	NGT Insulin $2^{d\text{-}phase}$ Pearson	NGT C-peptide $2^{d\text{-}phase}$ Spearman	NGT C-peptide $2^{d\text{-}phase}$ Pearson	IFG/IGT Insulin $1^{st\text{-}phase}$ Spearman	IFG/IGT Insulin $1^{st\text{-}phase}$ Pearson	IFG/IGT C-peptide $1^{st\text{-}phase}$ Spearman	IFG/IGT C-peptide $1^{st\text{-}phase}$ Pearson	IFG/IGT Insulin $2^{d\text{-}phase}$ Spearman	IFG/IGT Insulin $2^{d\text{-}phase}$ Pearson	IFG/IGT C-peptide $2^{d\text{-}phase}$ Spearman	IFG/IGT C-peptide $2^{d\text{-}phase}$ Pearson
BIGTT-AIR$_{0\text{-}30\text{-}120}$	0.692***	0.919***	0.621***	0.868***	0.586***	0.855***	0.43**	0.81***	0.634**	0.851***	0.626**	0.868***	0.617**	0.874***	0.632**	0.853***
BIGTT-AIR$_{0\text{-}60\text{-}120}$	0.576***	0.718***	0.472***	0.711***	0.53***	0.701***	0.357*	0.663***	0.78***	0.8***	0.745***	0.828***	0.736***	0.823***	0.73***	0.807***
CIR$_{120}$	0.526***	0.638***	0.382*	0.508***	0.531***	0.573***	0.445**	0.502***	0.601**	0.33	0.633**	0.42	0.581**	0.462*	0.578**	0.479*
CIR$_{30}$	0.615***	0.695***	0.555***	0.63***	0.626***	0.542***	0.51***	0.566***	0.67***	0.725***	0.653***	0.71***	0.608**	0.739***	0.651**	0.663***
CIR$_{60}$	0.45**	0.387**	0.353*	0.229	0.523***	0.397**	0.398**	0.366*	0.575**	-0.061	0.523*	-0.095	0.565**	-0.067	0.533**	-0.085
CP$_{120}$/CP$_0$	-0.184	-0.192	-0.215	-0.307*	-0.061	-0.244	-0.2	-0.279	-0.036	-0.236	0.106	-0.112	-0.135	-0.27	0.099	0.016
CP$_{30}$/CP$_0$	-0.044	0.076	-0.009	0.011	-0.03	-0.055	-0.08	-0.108	0.234	0.017	0.212	0.111	0.155	-0.037	0.209	0.171
CP$_{60}$/CP$_0$	-0.037	-0.063	0.001	-0.162	0.014	-0.104	-0.009	-0.143	0.091	-0.119	0.051	-0.061	0.09	-0.154	0.046	0.003
CP$_0$	0.412**	0.415**	0.457**	0.58***	0.454**	0.537***	0.516***	0.572***	0.541**	0.731***	0.524*	0.749***	0.433*	0.775***	0.324	0.667***
CP$_0$/G$_0$	0.513***	0.661***	0.535***	0.681***	0.466***	0.657***	0.525***	0.703***	0.649**	0.723***	0.628**	0.76***	0.484*	0.742***	0.411	0.67***
CP$_{120}$	0.215	0.449**	0.219	0.31*	0.419**	0.355*	0.352*	0.44**	0.452*	0.229	0.57**	0.339	0.249	0.234	0.405	0.401

Fasting/OGTT -based indices	Hyperglycaemic clamp – derived measurements															
	**NGT Insulin $1^{st\text{-}phase}$ Spearman**	**NGT Insulin $1^{st\text{-}phase}$ Pearson**	**NGT C-peptide $1^{st\text{-}phase}$ Spearman**	**NGT C-peptide $1^{st\text{-}phase}$ Pearson**	**NGT Insulin $2^{d\text{-}phase}$ Spearman**	**NGT Insulin $2^{d\text{-}phase}$ Pearson**	**NGT C-peptide $2^{d\text{-}phase}$ Spearman**	**NGT C-peptide $2^{d\text{-}phase}$ Pearson**	**IFG/IGT Insulin $1^{st\text{-}phase}$ Spearman**	**IFG/IGT Insulin $1^{st\text{-}phase}$ Pearson**	**IFG/IGT C-peptide $1^{st\text{-}phase}$ Spearman**	**IFG/IGT C-peptide $1^{st\text{-}phase}$ Pearson**	**IFG/IGT Insulin $2^{d\text{-}phase}$ Spearman**	**IFG/IGT Insulin $2^{d\text{-}phase}$ Pearson**	**IFG/IGT C-peptide $2^{d\text{-}phase}$ Spearman**	**IFG/IGT C-peptide $2^{d\text{-}phase}$ Pearson**
CP_{120}/G_{120}	0.46**	0.612***	0.517***	0.499***	0.498***	0.477***	0.57***	0.578***	0.401	0.129	0.508*	0.2	0.25	0.158	0.412	0.25
CP_{30}	0.411**	0.672***	0.465***	0.661***	0.503***	0.592***	0.509***	0.587***	0.764***	0.722***	0.718***	0.785***	0.631**	0.726***	0.535*	0.723***
CP_{30}/G_{30}	0.43**	0.654***	0.462***	0.546***	0.463**	0.431**	0.43**	0.517***	0.742***	0.751***	0.742***	0.789***	0.612**	0.763***	0.636**	0.721***
CP_{60}	0.437**	0.579***	0.524***	0.533***	0.605***	0.554***	0.636***	0.608***	0.613**	0.541**	0.581**	0.604**	0.51*	0.543**	0.411	0.568**
CP_{60}/G_{60}	0.492***	0.638***	0.524***	0.505***	0.539***	0.428**	0.546***	0.508***	0.504*	0.559**	0.508*	0.565**	0.397	0.561**	0.382	0.493*
HOMA-%B	0.577***	0.616***	0.407**	0.686***	0.48***	0.692***	0.318*	0.563***	0.688***	0.733***	0.608**	0.784***	0.66***	0.766***	0.552**	0.81***
HOMA2-%B(CP)	0.517***	0.658***	0.51***	0.656***	0.403**	0.6***	0.42**	0.581***	0.704***	0.59**	0.718***	0.665***	0.519*	0.563**	0.537*	0.58**
HOMA2-%B(I)	0.49**	0.668***	0.494**	0.751***	0.429**	0.727***	0.401*	0.709***	0.678***	0.706***	0.574**	0.76***	0.64**	0.734***	0.52*	0.787***
I_0	0.473***	0.39**	0.338*	0.568***	0.542***	0.586***	0.391**	0.47***	0.618**	0.769***	0.511*	0.8***	0.621**	0.842***	0.463*	0.832***
I_0/G_0	0.498***	0.475***	0.349*	0.632***	0.539***	0.645***	0.38**	0.499***	0.695***	0.771***	0.607**	0.808***	0.678***	0.831***	0.548**	0.837***
I_{120}	0.341*	0.645***	0.286*	0.53***	0.585***	0.611***	0.37**	0.631***	0.633**	0.399	0.636**	0.529**	0.541**	0.466*	0.509*	0.643***
I_{120}/G_{120}	0.475***	0.755***	0.397**	0.65***	0.686***	0.721***	0.518***	0.733***	0.623**	0.437*	0.615**	0.567**	0.547**	0.531**	0.517*	0.68***

**Fasting/OGTT -based indices**	**Hyperglycaemic clamp – derived measurements**															
	**NGT Insulin $1^{st-phase}$ Spearman**	**NGT Insulin $1^{st-phase}$ Pearson**	**NGT C-peptide $1^{st-phase}$ Spearman**	**NGT C-peptide $1^{st-phase}$ Pearson**	**NGT Insulin $2^{d-phase}$ Spearman**	**NGT Insulin $2^{d-phase}$ Pearson**	**NGT C-peptide $2^{d-phase}$ Spearman**	**NGT C-peptide $2^{d-phase}$ Pearson**	**IFG/ IGT Insulin $1^{st-phase}$ Spearman**	**IFG/ IGT Insulin $1^{st-phase}$ Pearson**	**IFG/ IGT C-peptide $1^{st-phase}$ Spearman**	**IFG/ IGT C-peptide $1^{st-phase}$ Pearson**	**IFG/ IGT Insulin $2^{d-phase}$ Spearman**	**IFG/ IGT Insulin $2^{d-phase}$ Pearson**	**IFG/ IGT C-peptide $2^{d-phase}$ Spearman**	**IFG/ IGT C-peptide $2^{d-phase}$ Pearson**
I_{30}	0.706***	0.902***	0.641***	0.871***	0.704***	0.901***	0.595***	0.718***	0.669***	0.775***	0.6**	0.813***	0.58**	0.807***	0.545**	0.81***
ΔI_{30}	0.694***	0.882***	0.626***	0.85***	0.684***	0.881***	0.571***	0.689***	0.63**	0.753***	0.593**	0.79***	0.557**	0.775***	0.552**	0.781***
I_{30}/G_{30}	0.742***	0.901***	0.663***	0.885***	0.732***	0.882***	0.587***	0.77***	0.676***	0.83***	0.648***	0.845***	0.587**	0.855***	0.624**	0.818***
I_{60}	0.507***	0.496***	0.454**	0.629***	0.656***	0.683***	0.543***	0.594***	0.561**	0.555**	0.503*	0.637**	0.536**	0.622**	0.433*	0.682***
ΔI_{60}	0.49***	0.48***	0.454**	0.614***	0.651***	0.671***	0.545***	0.577***	0.537**	0.522*	0.477*	0.607**	0.503*	0.586**	0.403	0.653***
I_{60}/G_{60}	0.612***	0.722***	0.542***	0.755***	0.725***	0.8***	0.606***	0.714***	0.645**	0.698***	0.577**	0.753***	0.593**	0.753***	0.509*	0.763***
IGI_{120}	0.538***	0.534***	0.532**	0.483**	0.578***	0.534***	0.564***	0.458**	0.219	0.156	0.256	0.229	0.273	0.233	0.319	0.371
IGI_{30}	0.632***	0.678***	0.584***	0.641***	0.683***	0.583***	0.569***	0.595***	0.625**	0.461*	0.615**	0.437*	0.534**	0.466*	0.602**	0.407
IGI_{60}	0.42**	0.234	0.401**	0.255	0.617***	0.332*	0.546***	0.356*	0.723***	0.426*	0.641***	0.453*	0.733***	0.555**	0.6**	0.414*
I_{120}/I_0	-0.051	0.15	-0.021	0.015	0.137	0.085	0.058	0.104	0.146	-0.08	0.205	0.031	0.04	-0.111	0.077	0.066
I_{30}/I_0	0.134	0.364*	0.263	0.41**	0.146	0.335*	0.189	0.304*	0.268	0.064	0.268	0.089	0.213	0.037	0.28	0.081
I_{60}/I_0	0.115	0.077	0.181	0.1	0.263	0.162	0.264	0.133	0.166	-0.007	0.141	0.057	0.132	-0.025	0.093	0.064

Fasting/OGTT-based indices	Hyperglycaemic clamp – derived measurements															
	**NGT Insulin 1st-phase Spearman**	**NGT Insulin 1st-phase Pearson**	**NGT C-peptide 1st-phase Spearman**	**NGT C-peptide 1st-phase Pearson**	**NGT Insulin 2d-phase Spearman**	**NGT Insulin 2d-phase Pearson**	**NGT C-peptide 2d-phase Spearman**	**NGT C-peptide 2d-phase Pearson**	**IFG/IGT Insulin 1st-phase Spearman**	**IFG/IGT Insulin 1st-phase Pearson**	**IFG/IGT C-peptide 1st-phase Spearman**	**IFG/IGT C-peptide 1st-phase Pearson**	**IFG/IGT Insulin 2d-phase Spearman**	**IFG/IGT Insulin 2d-phase Pearson**	**IFG/IGT C-peptide 2d-phase Spearman**	**IFG/IGT C-peptide 2d-phase Pearson**
Kadowaki model	0.702***	0.875***	0.627***	0.861***	0.696***	0.858***	0.542***	0.737***	0.636**	0.803***	0.608**	0.817***	0.543**	0.82***	0.567**	0.785***
$\log(I_0)$	0.504***	0.481***	0.377**	0.534***	0.569***	0.571***	0.427**	0.524***	0.618**	0.705***	0.511*	0.746***	0.621**	0.766***	0.463*	0.759***
$\log(I_{120})$	0.341*	0.466***	0.286*	0.4**	0.585***	0.515***	0.37**	0.488***	0.633**	0.471*	0.636**	0.586**	0.541**	0.497*	0.509*	0.612**
$\log(I_{30})$	0.706***	0.794***	0.641***	0.804***	0.704***	0.809***	0.595***	0.705***	0.669***	0.651***	0.6**	0.705***	0.58**	0.679***	0.545**	0.701***
$\log(I_{60})$	0.507***	0.456**	0.454**	0.538***	0.656***	0.604***	0.543***	0.553***	0.561**	0.47*	0.503*	0.509*	0.536**	0.505*	0.433*	0.499*
$\log(I_{90})$	0.35*	0.464***	0.317*	0.46***	0.564***	0.539***	0.468***	0.557***	0.404	0.289	0.357	0.381	0.425*	0.37	0.265	0.412
CPI_0	0.513***	0.661***	0.535***	0.681***	0.466***	0.657***	0.525***	0.703***	0.649**	0.723***	0.628**	0.76***	0.484*	0.742***	0.411	0.67***
CPI_{120}	0.46**	0.612***	0.517***	0.499***	0.498***	0.477***	0.57***	0.578***	0.401	0.129	0.508*	0.2	0.25	0.158	0.412	0.25
first-phase Stumvoll	0.701***	0.859***	0.636***	0.867***	0.717***	0.87***	0.577***	0.761***	0.644**	0.796***	0.617**	0.818***	0.58**	0.846***	0.574**	0.827***
second-phase Stumvoll	0.658***	0.864***	0.576***	0.87***	0.675***	0.905***	0.581***	0.732***	0.615**	0.772***	0.559**	0.809***	0.548**	0.814***	0.468*	0.812**

Supplementary Table 3: Correlation between fasting and OGTT-derived insulin secretion indices and GLP-1-stimulated insulin (C-peptide) secretion

	NGT-group(n=49).				IFG and/or IGT -group (n=23).			
Indices	Insulin GLP1 Spearman	C-peptide GLP1 Spearman	Insulin GLP1 Pearson	C-peptide GLP1 Pearson	Insulin GLP1 Spearman	C-peptide GLP1 Spearman	Insulin GLP1 Pearson	C-peptide GLP1 Pearson
AUC (CP_{0-120})/AUC (G_{0-120})	0.525 ***	0.38 **	0.511 ***	0.45 **	0.652 **	0.412	0.669 ***	0.523 *
AUC(CP_{0-30})/AUC(G_{0-30})	0.509 ***	0.395 **	0.607 ***	0.565 ***	0.736 ***	0.557 **	0.768 ***	0.624 **
AUC (CP_{0-60})/AUC (G_{0-60})	0.541 ***	0.392 **	0.546 ***	0.483 ***	0.651 **	0.374	0.685 ***	0.541 **
AUC(CP_{all})	0.484 ***	0.343 *	0.526 ***	0.48 ***	0.49 *	0.241	0.545 **	0.454 *
AUC(I_{all})	0.613 ***	0.414 **	0.79 ***	0.638 ***	0.483 *	0.269	0.681 ***	0.573 **
AUC(I_{0-120})/AUC(G_{0-120})	0.723 ***	0.529 ***	0.789 ***	0.61 ***	0.664 ***	0.414	0.75 ***	0.615 **
AUC(I_{0-30})/AUC(G_{0-30})	0.728 ***	0.54 ***	0.775 ***	0.57 ***	0.725 ***	0.517 *	0.799 ***	0.706 ***
AUC(I_{0-60})/AUC(G_{0-60})	0.649 ***	0.441 **	0.756 ***	0.59 ***	0.588 **	0.327	0.717 ***	0.587 **
BIGTT-$AIR_{0-30-120}$	0.631 ***	0.466 ***	0.715 ***	0.6 ***	0.66 ***	0.49 *	0.771 ***	0.675 ***
BIGTT-$AIR_{0-60-120}$	0.599 ***	0.376 **	0.761 ***	0.703 ***	0.682 ***	0.519 *	0.741 ***	0.619 **
CIR_{120}	0.435 **	0.32 *	0.378 *	0.266	0.608 **	0.38	0.529 *	0.318
CIR_{30}	0.669 ***	0.523 ***	0.545 ***	0.41 **	0.777 ***	0.642 **	0.702 ***	0.61 **
CIR_{60}	0.51 ***	0.335 *	0.123	-0.037	0.758 ***	0.427 *	0.063	0.157
CP_{120}/CP_0	-0.252	-0.372 **	-0.352 *	-0.364 *	-0.119	0.033	-0.169	0.047
CP_{30}/CP_0	-0.031	-0.038	-0.077	-0.107	0.238	0.317	0.048	0.332
CP_{60}/CP_0	-0.05	-0.103	-0.161	-0.207	-0.005	0.015	-0.123	0.076
CP_0	0.361 *	0.304 *	0.496 ***	0.369 *	0.489 *	0.19	0.691 ***	0.436 *
CP_0/G_0	0.398 **	0.357 *	0.678 ***	0.691 ***	0.516 *	0.269	0.677 ***	0.464 *
CP_{120}	0.182	-0.023	0.32 *	0.321 *	0.292	0.234	0.312	0.303
CP_{120}/G_{120}	0.386 **	0.29 *	0.409 **	0.415 **	0.5 *	0.321	0.252	0.185
CP_{30}	0.445 **	0.355 *	0.568 ***	0.494 ***	0.606 **	0.456 *	0.685 ***	0.602 **
CP_{30}/G_{30}	0.506 ***	0.397 **	0.507 ***	0.455 **	0.765 ***	0.617 **	0.75 ***	0.63 **
CP_{60}	0.512 ***	0.387 **	0.522 ***	0.453 **	0.431 *	0.228	0.51 *	0.437 *
CP_{60}/G_{60}	0.539 ***	0.38 **	0.445 **	0.371 **	0.595 **	0.334	0.608 **	0.489 *
HOMA-%B	0.431 **	0.304 *	0.674 ***	0.59 ***	0.565 **	0.349	0.755 ***	0.649 ***
HOMA2-%B(CP)	0.323 *	0.291 *	0.552 ***	0.531 ***	0.582 **	0.396	0.553 **	0.457 *
HOMA2-%B(I)	0.34 *	0.344 *	0.685 ***	0.668 ***	0.543 *	0.296	0.729 ***	0.634 **
I_0	0.471 ***	0.29 *	0.598 ***	0.379 **	0.544 **	0.262	0.809 ***	0.647 ***
I_0/G_0	0.48 ***	0.304 *	0.623 ***	0.407 **	0.582 **	0.329	0.805 ***	0.66 ***
I_{120}	0.411 **	0.174	0.583 ***	0.542 ***	0.339	0.358	0.584 **	0.561 **
I_{120}/G_{120}	0.528 ***	0.314 *	0.64 ***	0.568 ***	0.529 *	0.416 *	0.656 ***	0.587 **
I_{30}	0.663 ***	0.519 ***	0.734 ***	0.507 ***	0.642 **	0.488 *	0.759 ***	0.689 ***
ΔI_{30}	0.637 ***	0.498 ***	0.689 ***	0.449 **	0.609 **	0.487 *	0.726 ***	0.676 ***
I_{30}/G_{30}	0.747 ***	0.567 ***	0.757 ***	0.548 ***	0.726 ***	0.556 **	0.792 ***	0.698 ***

	NGT-group(n=49).				IFG and/or IGT -group (n=23).			
**Indices**	**Insulin GLP1 Spearman**	**C-peptide GLP1 Spearman**	**Insulin GLP1 Pearson**	**C-peptide GLP1 Pearson**	**Insulin GLP1 Spearman**	**C-peptide GLP1 Spearman**	**Insulin GLP1 Pearson**	**C-peptide GLP1 Pearson**
I_{60}	0.556 ***	0.362 *	0.721 ***	0.579 ***	0.411	0.204	0.641 ***	0.527 **
ΔI_{60}	0.55 ***	0.359 *	0.697 ***	0.544 ***	0.363	0.186	0.611 **	0.504 *
I_{60}/G_{60}	0.696 ***	0.498 ***	0.748 ***	0.561 ***	0.627 **	0.363	0.744 ***	0.6 **
IGI_{120}	0.568 ***	0.541 ***	0.295	0.247	0.358	0.155	0.374	0.316
IGI_{30}	0.743 ***	0.578 ***	0.583 ***	0.413 **	0.718 ***	0.581 **	0.484 *	0.473 *
IGI_{60}	0.524 ***	0.344 *	0.197	0.098	0.6 **	0.235	0.665 ***	0.184
I_{120}/I_0	-0.017	-0.119	-0.078	-0.137	-0.113	0.089	-0.073	0.04
I_{30}/I_0	0.13	0.162	0.206	0.112	0.341	0.343	0.064	0.199
I_{60}/I_0	0.198	0.121	0.124	0.031	0.042	-0.053	-0.034	0.022
Kadowaki model	0.701 ***	0.527 ***	0.707 ***	0.486 ***	0.686 ***	0.533 **	0.755 ***	0.681 ***
$\log(I_0)$	0.503 ***	0.333 *	0.644 ***	0.556 ***	0.544 **	0.262	0.75 ***	0.593 **
$\log(I_{120})$	0.411 **	0.174	0.461 ***	0.319 *	0.339	0.358	0.509 *	0.507 *
$\log(I_{30})$	0.663 ***	0.519 ***	0.737 ***	0.56 ***	0.642 **	0.488 *	0.684 ***	0.634 **
$\log(I_{60})$	0.556 ***	0.362 *	0.627 ***	0.489 ***	0.411	0.204	0.481 *	0.359
$\log(I_{90})$	0.319 *	0.185	0.454 **	0.384 **	0.339	0.11	0.461 *	0.335
CPI_0	0.398 **	0.357 *	0.678 ***	0.691 ***	0.516 *	0.269	0.677 ***	0.464 *
CPI_{120}	0.386 **	0.29 *	0.409 **	0.415 **	0.5 *	0.321	0.252	0.185
first-phase Stumvoll	0.739 ***	0.573 ***	0.815 ***	0.617 ***	0.757 ***	0.545 **	0.831 ***	0.706 ***
second-phase Stumvoll	0.6 ***	0.468 ***	0.776 ***	0.582 ***	0.544 **	0.345	0.758 ***	0.665 ***

Supplementary Table 4: Fasting- and OGTT-derived measures of beta cell function

Fasting/OGTT-based indices	Formula
AUC (CP_{0-120})/AUC (G_{0-120})	AUC for insulin, glucose and C-peptide were calculated with the trapezoid method
AUC(CP_{0-30})/AUC(G_{0-30})	
AUC (CP_{0-60})/AUC (G_{0-60})	
AUC(CP_{all})	
AUC(I_{all})	
AUC(I_{0-120})/AUC(G_{0-120})	
AUC(I_{0-30})/AUC(G_{0-30})	
AUC(I_{0-60})/AUC(G_{0-60})	
BIGTT-$AIR_{0-30-120}$	exp[8.20 + (0.00178 × I_0) + (0.00168 × I_{30}) - (0.000383 × I_{120}) - (0.314 × G_0)-(0.109 × G_{30}) + (0.0781 × G_{120}) + (0.180 ×sex) + (0.032 × BMI)]
BIGTT-$AIR_{0-60-120}$	exp[8.20 + (0.00178 × I_0) + (0.00168 × I_{60}) - (0.000383 × I_{120}) - (0.314 × G_0)-(0.109 × G_{60}) + (0.0781 × G_{120}) + (0.180 ×sex) + (0.032 × BMI)]
CIR_{120}	I_{120} /(G_{120} x (G_{120} – 3.89))
CIR_{30}	I_{30} /(G_{30} x (G_{30} – 3.89))
CIR_{60}	I_{60} /(G_{60} x (G_{60} – 3.89))
CP_{120}/CP_0	CP_{120}/CP_0
CP_{30}/CP_0	CP_{30}/CP_0
CP_{60}/CP_0	CP_{60}/CP_0
CP_0	
CP_0/G_0	CP_0/G_0
CP_{120}	
CP_{120}/G_{120}	CP_{120}/G_{120}
CP_{30}	
CP_{30}/G_{30}	CP_{30}/G_{30}
CP_{60}	
CP_{60}/G_{60}	CP_{60}/G_{60}
HOMA-%B	(20 × I_0)/(G_0– 3.5)
HOMA2-%B(CP)	https://www.dtu.ox.ac.uk/homacalculator/
HOMA2-%B(I)	https://www.dtu.ox.ac.uk/homacalculator/
I_0	
I_0/G_0	I_0/G_0
I_{120}	
I_{120}/G_{120}	I_{120}/G_{120}
I_{30}	
ΔI_{30}	I_{30} - I_0
I_{30}/G_{30}	I_{30}/G_{30}
I_{60}	
ΔI_{60}	I_{60} - I_0
I_{60}/G_{60}	I_{60}/G_{60}
IGI_{120}	(I_{120} – I_0)/(G_{120} – G_0
IGI_{30}	(I_{30} – I_0)/(G_{30} – G_0)
IGI_{60}	(I_{60} – I_0)/(G_{60} – G_0)
I_{120}/I_0	I_{120}/I_0

I_{30}/I_0	I_{30}/I_0
I_{60}/I_0	I_{60}/I_0
Kadowaki model	$(I_{30} - I_0)/(G_{30} - G_0)$
$\log(I_0)$	$\log(I_0)$
$\log(I_{120})$	$\log(I_{120})$
$\log(I_{30})$	$\log(I_{30})$
$\log(I_{60})$	$\log(I_{60})$
$\log(I_{90})$	$\log(I_{90})$
CPI_0	$100 \times CP_0(ng/mL)/G_0(mg/dL)$
CPI_{120}	$100 \times CP_{120}(ng/mL)/G_{120}(mg/dL)$
first-phase Stumvoll	$1283 + 1.829 \times I_{30} – 138.7 \times G_{30} + 3.772 \times I_0$
second-phase Stumvoll	$286 + 0.416 \times I_{30} – 25.94 \times G_{30} + 0.926 \times I_0$

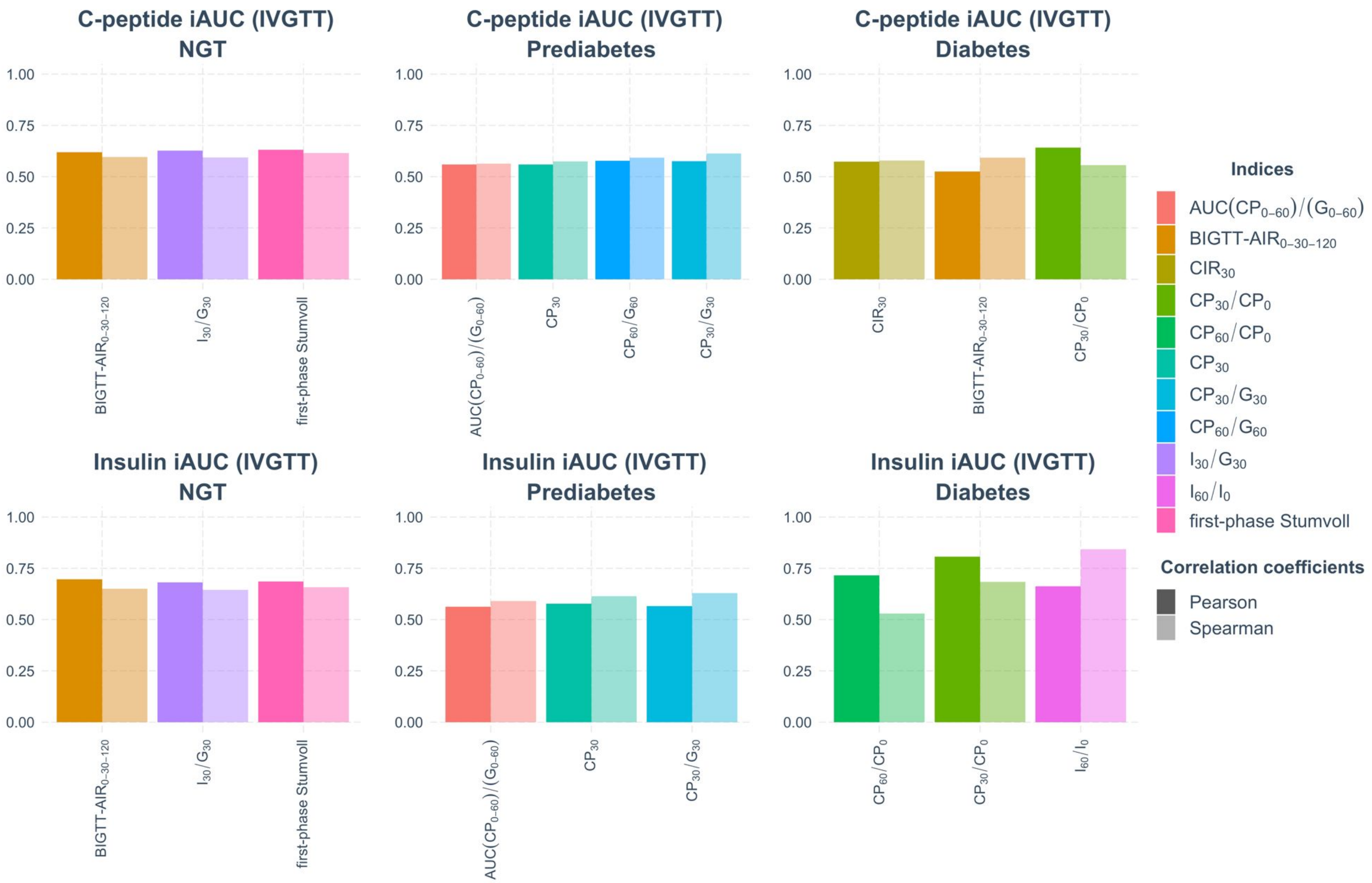

Supplementary Figure 1: Top-3 indices by Spearman's and Pearson's correlation coefficients in subsets with T2D, prediabetes and NGT compared to first-phase IVGTT

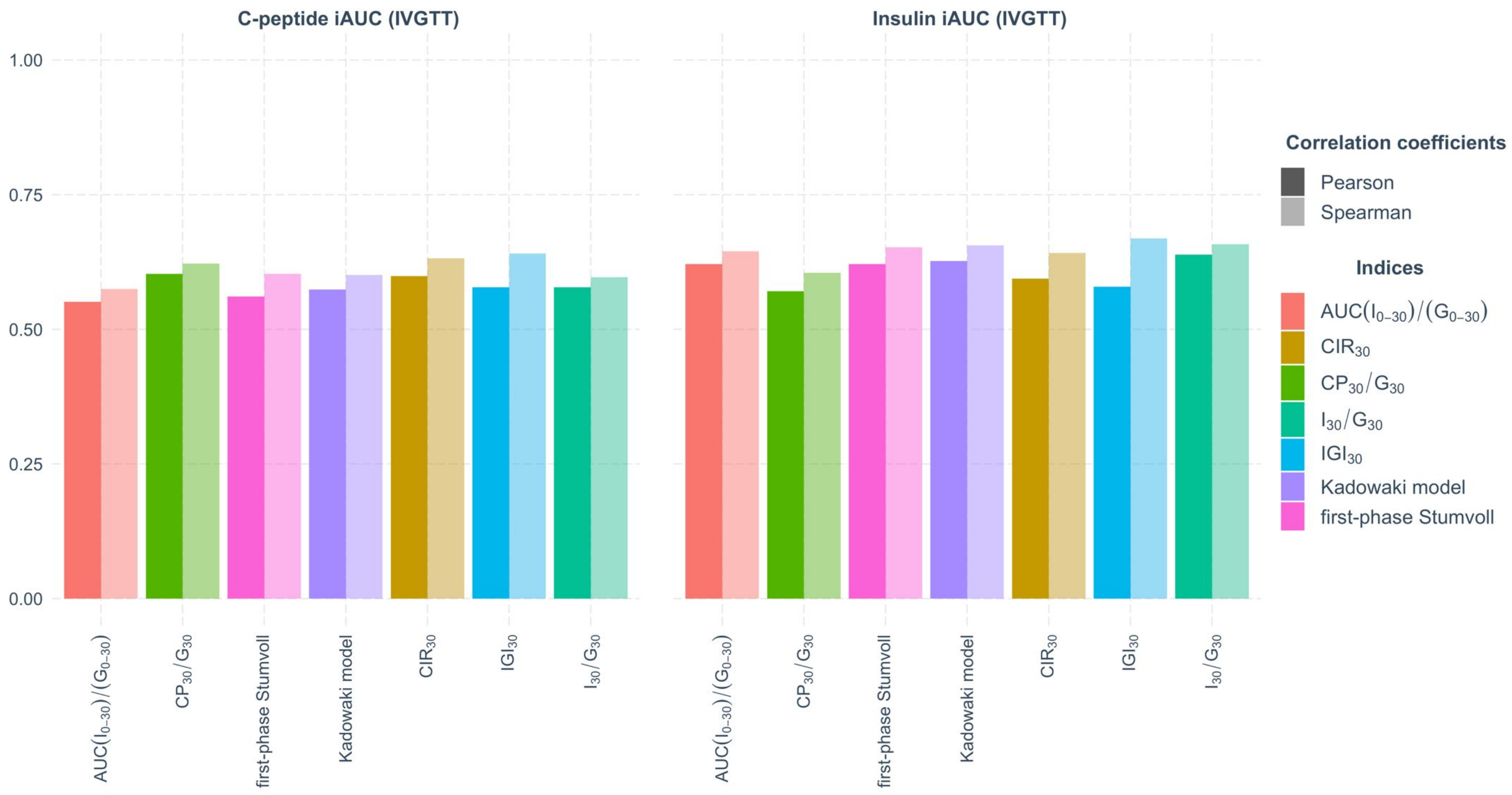

Supplementary Figure 2: Top indices by Spearman's and Pearson's correlation coefficients in the entire cohort (n=316) compared to first-phase IVGTT

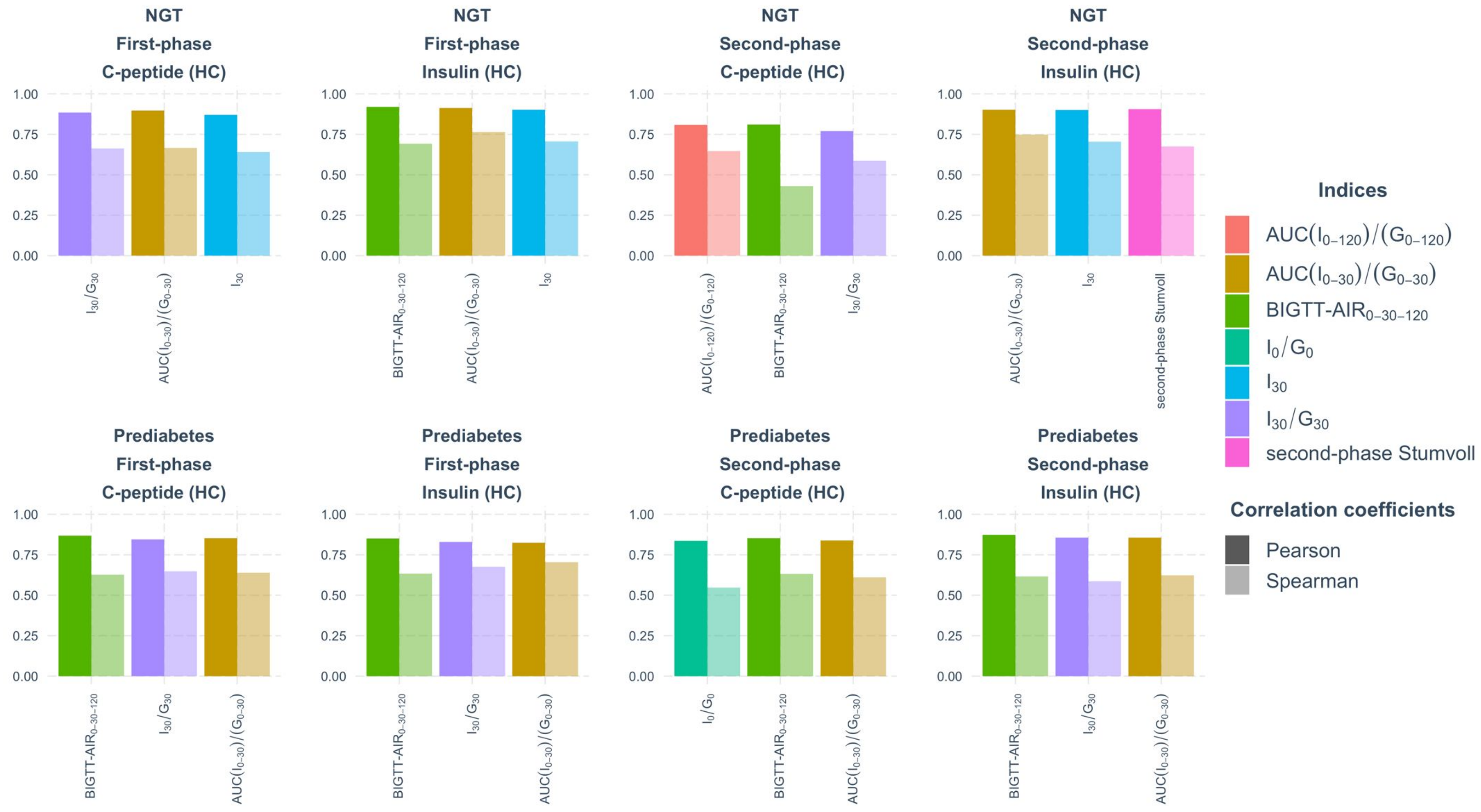

Supplementary Figure 3: Top-indices by Spearman's and Pearson's correlation coefficients in NGT and prediabetes groups compared to first- and second phase hyperglycaemic clamp

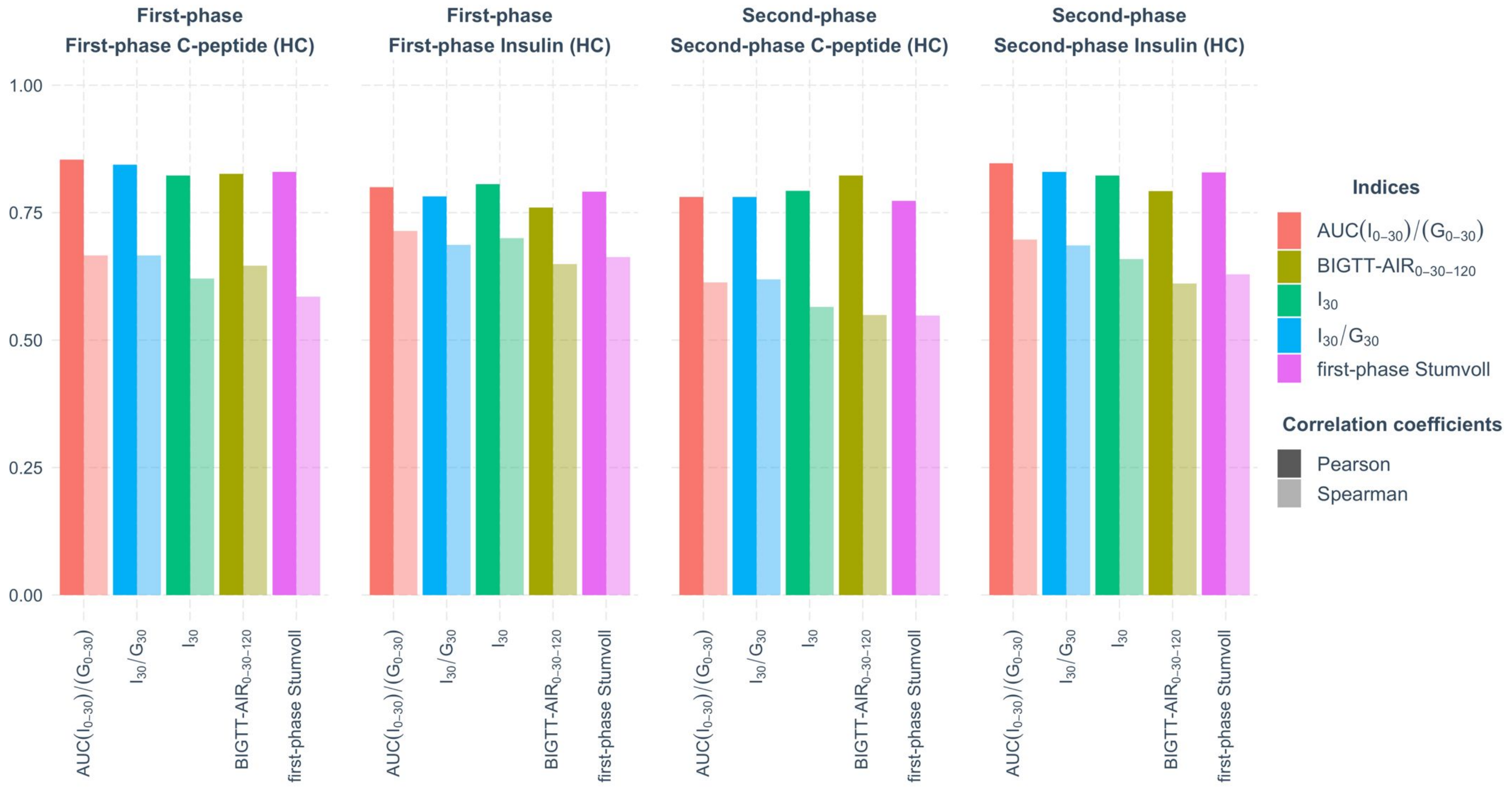

Supplementary Figure 4: Top-indices by Spearman's and Pearson's correlation coefficients in the whole group (n=76) compared to first- and second phase of hyperglycaemic clamp
